# Dysregulation of microRNAs in the Brains of Mice Infected with Powassan Virus

**DOI:** 10.3390/v17101288

**Published:** 2025-09-23

**Authors:** Amany Elsharkawy, Komal Arora, Hamid Reza Jahantigh, Mukesh Kumar

**Affiliations:** Department of Biology, College of Arts and Sciences, Georgia State University, Atlanta, GA 30303, USA; aelsharkawy2@gsu.edu (A.E.); karora@gsu.edu (K.A.); hjahantigh@gsu.edu (H.R.J.)

**Keywords:** flavivirus, Powassan virus, microRNAs, neuroinflammation

## Abstract

microRNAs (miRNAs) are known to play critical roles in the regulation of gene expression during neurodegenerative diseases and neurotropic viral infections. However, their specific contribution to the pathogenesis of Powassan virus (POWV) infection in the brain remains poorly understood. Understanding miRNA dynamics in the brain during POWV infection may reveal novel insights into viral neuropathogenesis and host antiviral responses. Therefore, in the present study, we analyzed miRNA expression profiles in the mouse brain at different time points following a peripheral POWV infection. A total of 599 miRNAs were examined at day 3, 6, and 9 post-infection. Infection with POWV resulted in the modulation of several miRNAs in the brain at all time points. There was a progressive increase in the number of dysregulated miRNAs over the course of infection. This correlated with POWV dissemination into the brain with a progressive increase in viral RNA levels that peaked at day 9 post-infection. There was an early upregulation of miR-1983, miR-19a, and miR-216b that persisted until day 9 post-infection. POWV infection also resulted in the downregulation of miR-500 at all examined time points. Using IPA, we determined the significant canonical pathways affected by miRNA dysregulation. POWV infection modulated the activation of the thyroid hormone receptor and retinoid X receptor (TR/RXR) and the regulation of the phosphatase and tensin homolog (PTEN). Additionally, macrophage classical activation and growth arrest and DNA damage-inducible 45 (GADD45) signaling were activated as early as day 3 post-infection and persisted until day 9 post-infection. Furthermore, our analysis revealed the activation of cell death pathways such as necrosis and apoptosis and the inhibition of cell cycle progression, as well as leukopoiesis. To our knowledge, this is the first study to evaluate the modulation of miRNAs in the brain following POWV infection.

## 1. Introduction

Powassan virus (POWV) is a neurotropic virus that belongs to the family orthoflavivirus [1,2,3]. Unlike closely related viruses such as West Nile Virus (WNV) and Japanese encephalitis virus (JEV) that are transmitted through mosquitos, POWV is a tick-borne virus transmitted through *Ixodes scapularis* ticks [4]. POWV is native to North America and was first discovered in Powassan, Ontario, following a severe encephalitis case in 1958 [4]. POWV causes symptoms that can range between mild and severe manifestations including encephalitis and meningitis. Approximately 10% of POWV cases are fatal, and 50% of surviving patients suffer from long-term neurological sequelae. Recently, POWV has attracted increased attention due to rising case counts [2,3]. Despite the gravity of the disease caused by POWV, there are no available vaccines or therapeutics against POWV.

MicroRNAs (miRNAs) are non-coding RNAs approximately 20–25 nucleotides in length. miRNAs govern the expression of approximately 50% of protein-coding genes through post-transcriptional regulation of gene expression. By binding to the 3′ untranslated regions (3′ UTRs) of target messenger RNAs (mRNAs), miRNAs promote target mRNA degradation. These small non-coding RNAs fine-tune several physiological processes such as proliferation and cell death. In addition to their regulatory roles under normal physiological conditions, modulation of miRNAs expression has been implicated in pathological states, including diseases such as Alzheimer’s disease and Parkinson’s disease, as well as viral infections [5,6]. The modulation of miRNAs plays a pivotal role in the host response to flavivirus infections. The role of miRNAs in the pathogenesis of Zika virus (ZIKV), WNV, dengue virus (DENV), and JEV was previously shown [7,8,9,10,11,12]. Additionally, modulation of specific miRNAs during a hantavirus infection was observed [13]. Therefore, miRNAs can function as biomarkers for disease progression and targeting them can represent a potential therapeutic strategy [14].

In the current study, we infected wildtype C57BL/6 mice with POWV subcutaneously and collected brain tissues at various time points. We examined the expression levels of 599 miRNAs in infected brain tissues using the nCounter system. We identified the significantly dysregulated miRNAs at all time points. Further, we identified common and time-specific modulation of miRNAs. Using IPA, we determined the significant canonical pathways and the diseases and biofunctions associated with the dysregulated miRNAs following POWV infection.

## 2. Materials and Methods

### 2.1. In Vivo Mouse Experiments

Animal studies were carried out in accordance with the recommendations of Institutional Animal Care and Use Committees (IACUC). The protocols were approved by the Georgia State University IACUC (Protocol number: A24041). The POWV LB strain was obtained from BEI resources (BEI Resources, NIAID, NIH: Powassan Virus, LB, NR-51181). The complete genome of the POWV LB strain has been sequenced (GenBank: L06436). Experiments involving infectious POWV were performed in the Animal Biosafety Level 3 laboratory. Virus inoculations were performed under anesthesia that was induced and maintained with isoflurane. Six-week-old C57BL/6 mice were inoculated with 1000 plaque-forming units (PFU) of POWV or PBS (mock) via the footpad route. Brain tissues were collected at days 3, 6, and 9 post-infection (*n* = 3/group) and flash-frozen in 2-Methyl butane on dry ice.

### 2.2. RNA Extraction

Frozen brain tissue was pounded and lysed in 600 μL RLT RNA extraction buffer (Qiagen, Redwood City, CA, USA) with 0.1% β-mercaptoethanol (β-ME). Lysates were loaded onto the QIAshredder homogenizer (Qiagen, Catalog# 79656, Redwood City, CA, USA), and RNA was extracted with the Qiagen RNeasy Plus Mini Kit (Qiagen, Catalog# 74136, Redwood City, CA, USA) and resuspended in RNAse-free water [15,16,17]. The RNA concentration was determined with a NanoDrop One instrument.

### 2.3. Viral Burden Quantification

The cDNA library was created using the iScriptTM cDNA synthesis kit (Biorad, Catalog# 1708891, Hercules, CA, USA). The cDNA was used for qPCR using SsoAdvanced™ Universal Probes Supermix (Biorad, Catalog# 1725281, Hercules, CA, USA) [18]. Viral RNA levels were measured with primers and probes specific to POWV, as shown in Table 1. Viral genome copies were calculated using a standard curve and expressed per µg of total RNA.

### 2.4. NanoString nCounter^®^ Gene Expression

For miRNA analysis, we used the nCounter^®^ Mouse miRNA Expression Panel (NanoString, Seattle, WA, USA, Catalog# CSO-MMIR15-12). Raw data was normalized using the geometric mean values of the top 100 miRNA expressed in each sample using nSolver Analysis Software 4.0 (NanoString), according to the manufacturer’s guidelines. At each time point, transcript quantification was performed for each POWV-infected sample and mock-infected controls. We used three individual animals at days 3, 6, and 9 post-infection and three mock control animals. The generated average of each group was compared to the average of the mock-infected animals (control). Next, significantly differentially expressed miRNAs were determined based on the cutoff of an absolute Log2 Fold change value greater than 0.75 and a *p*-value < 0.05 (Table S1). Volcano plots were generated using ggplot2 and ggrepel [16,19].

For heatmap analysis, we used Euclidean distance to quantify differences between miRNA expression profiles at 3, 6, and 9 dpi. Hierarchical clustering was conducted using the complete linkage method. We applied Z-score normalization (row scaling) to standardize gene expression data, allowing comparison across different miRNAs [19].

### 2.5. Ingenuity Pathway Analysis (IPA)

Pathway analyses were conducted using IPA 2025 [19,20] (Qiagen, Redwood City, CA, USA). IPA provided Z-score analyses for canonical pathways, diseases and biofunctions, and miRNAs associated with specific pathways. The networks analyses were also generated using IPA.

### 2.6. Multiplex Immunoassay

Brain tissues harvested from POWV-infected and mock-infected mice (*n* = 3 per time point) were homogenized in 1X PBS with protease inhibitor in the bullet blender (Next Advanced). Homogenates were tested using the MILLIPLEX MAP Mouse Cytokine/Chemokine Magnetic Bead Panel (Cat# MCYTMAG-70K-PX25) as per manufacturer instructions. We calculated the sample concentrations using the Belysa^®^ Immunoassay Curve Fitting Software (Millipore Sigma, version 1.2) [18,21].

### 2.7. Statistical Analysis

Statistical analyses for viral loads were performed using Prism 10. A *p*-value less than 0.05 was considered significant.

## 3. Results

### 3.1. Dysregulation of miRNAs in the Brain Following POWV Infection

**Figure 1:**
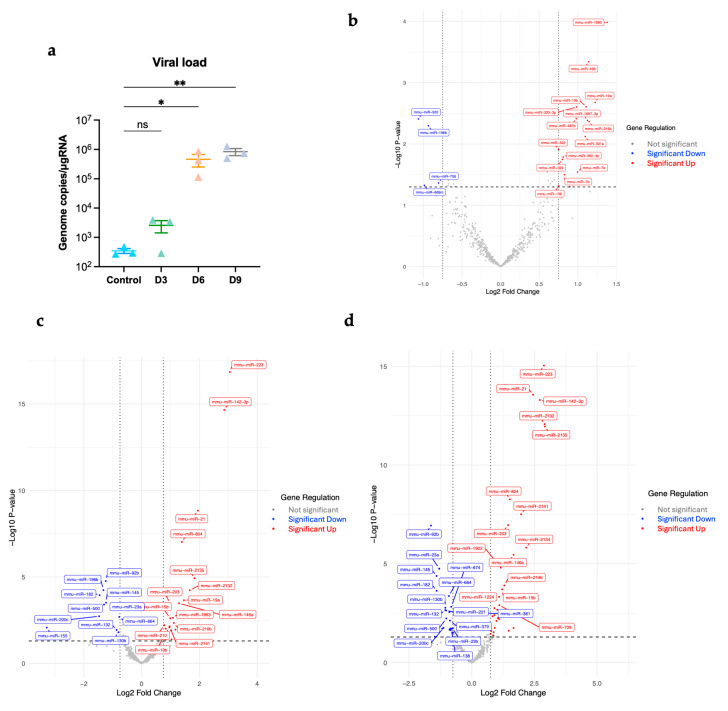
(**a**) Viral load in the brain was determined using RT-qPCR (*n* = 3 per time point). Statistical significance was determined with the Kruskal–Wallis test, followed by Dunn’s test; * *p* < 0.05; ** *p* < 0.01; ns, non-significant. Volcano plots (**b**) at day 3 post-infection, (**c**) at day 6 post-infection, (**d**) at day 9 post-infection. Significantly differentially expressed miRNAs were determined based on a cutoff *p*-value of less than 0.05 and absolute Log2 Fold change value greater than 0.75. Red represents significantly upregulated; blue represents significantly downregulated.

We infected six-week-old wild-type C57BL/6 mice subcutaneously with 1000 PFU of POWV or PBS (Mock). We euthanized mice (*n* = 3) at 3, 6, and 9 days post-infection (dpi). First, we performed RT-qPCR to quantify viral copy numbers in brains. We detected viral RNA in the brain as early as 3 dpi. Viral RNA levels increased significantly at 6 dpi. There was a progressive increase in viral RNA levels over the course of infection. The highest levels of viral RNA were detected in the brains at 9 dpi (Figure 1a).

A total of 599 miRNAs were examined in the infected brains at day 3, 6, and 9 post-infection. We identified the significantly differentially expressed miRNAs with an absolute Log2 fold change value greater than 0.75 and *p*-value less than 0.05. We generated volcano plots for each time point (Figure 1b–d).

**Figure 2:**
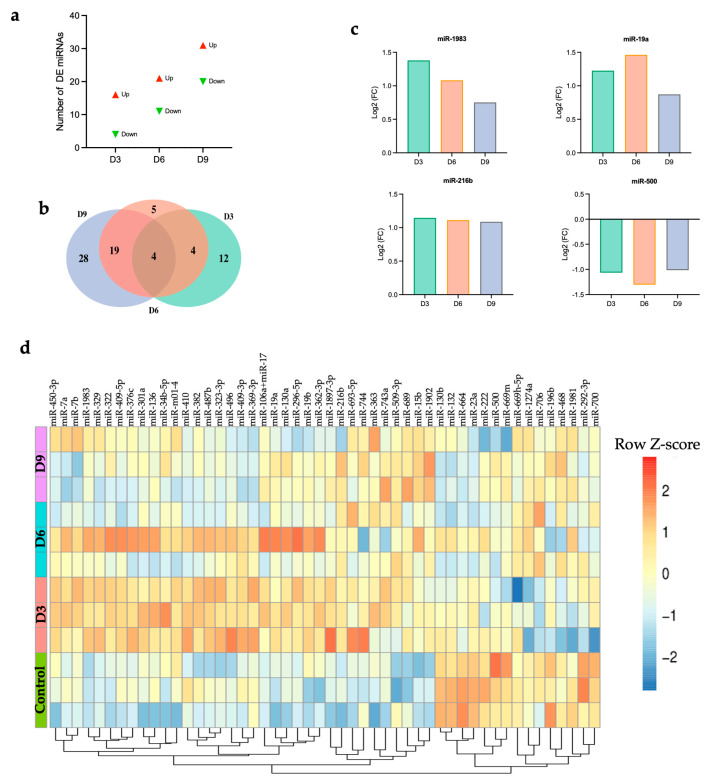
(**a**) Number of DE miRNAs at each time point, (**b**) Venn diagram, (**c**) common differentially expressed miRNAs, (**d**) heatmap showing hierarchical clustering of the top 50 DEGs at day 3, 6, and 9 post-infection for individual samples. Z-score normalization (row scaling) was applied to standardize the gene expression across samples. Each row represents an individual sample.

POWV infection induced a significant upregulation of 16, 21, and 31 genes and a significant downregulation of 4, 11, and 20 miRNAs at 3, 6, and 9 dpi, respectively (Figure 2a). There was a progressive increase in the number of significantly upregulated and downregulated miRNAs from 3 to 9 dpi (Table S1). The top significantly upregulated and downregulated miRNAs at 3, 6, and 9 dpi are shown in Table 2. Next, we generated Venn diagram to show overlapping miRNAs and time-specific miRNA (Figure 2b). Four miRNAs, miR-1983, miR-19a, miR-216b, and miR-500, were common among all the time points. We detected increased expression of miR-1983, miR-19a, and miR-216b at all time points. On the other hand, the expression of miR-500 was significantly downregulated at all time points (Figure 2c). We also identified time-specific miRNAs, shown in Table S2.

Next, we generated a heatmap of the top 50 miRNAs based on the highest expression variance across samples from the row variance of normalized counts for each time point to determine miRNA expression patterns across samples. Hierarchical clustering analysis identified two distinct clusters (Figure 2d). Cluster 1, characterized by enrichment of miRNAs such as miR-500 and miR-669m, demonstrated downregulation after infection with POWV compared to the control group. Cluster 2, characterized by the enrichment of miRNAs such as miR-15b and miR-1902, showed upregulation in the brain following POWV infection, especially at 9 dpi. Emerging evidence suggests the potential role of these miRNAs in regulating immune responses and antiviral defense mechanisms.

### 3.2. Network Analysis of Dysregulated miRNAs in POWV-Infected Brain

**Figure 3:**
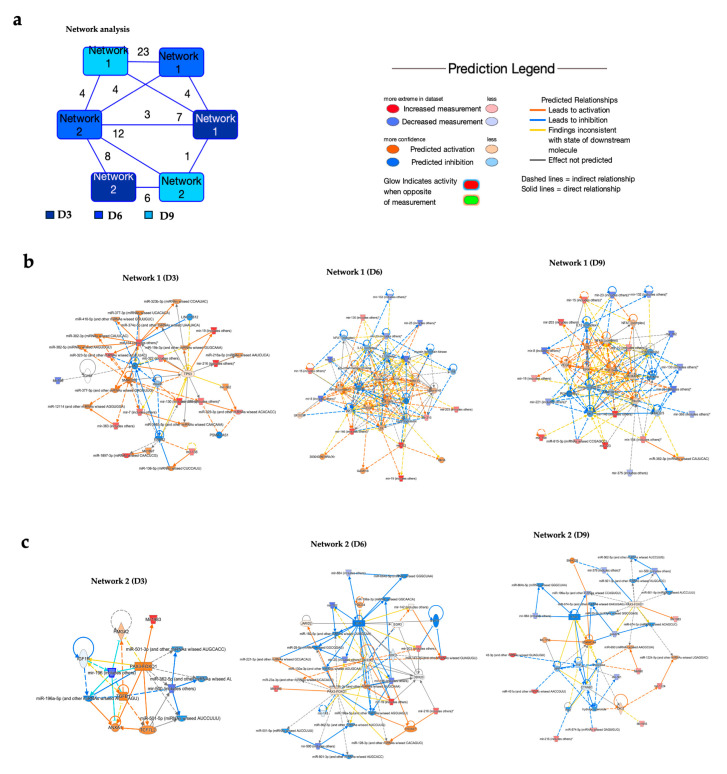
Network analysis of dysregulated miRNAs. (**a**) Overlapping networks. (**b**,**c**) Enriched networks at day 3, 6, and 9 post-infection. Red represents significantly upregulated; blue represents significantly downregulated. The color-coded arrows represent activation (orange), inhibition (blue), and unknown or indirect interactions (gray), with solid lines indicating confirmed interactions and dashed lines indicating predicted or inferred associations.

Using Ingenuity Pathway Analysis (IPA) software, we performed network analysis of dysregulated miRNAs (Table S3). Our analysis revealed two overlapping networks related to gene expression, cellular development, and tissue morphology (Figure 3a). These networks highlighted multiple miRNAs and their predicted or validated targets (Figure 3b). Network 1 illustrated pathways related to inflammation, immune response, and cell signaling. At day 3 post-infection, network 1 illustrated the interactions between specific miRNAs and key signaling molecules such as AKT, TP53, SMAD4, ERBB2, and insulin. Several of these modulated miRNAs, including miR-130, miR-136, miR-19, and miR-322, are shown to influence pathways involved in cell growth, apoptosis, and insulin signaling [22,23,24,25,26]. At day 6 post-infection, miRNAs such as miR-132, miR-15, miR-203, and miR-19 are shown to interact with central signaling molecules like NF-κB, IL-1β, VEGF, and ERK1/2. Particularly, miR-132 was a major inhibitory regulator, targeting several components involved in pro-inflammatory and proliferative signaling. This network analysis underscores the intricate control that miRNAs exert over cellular homeostasis and immune pathways following POWV infection. At day 9, network 1 illustrated a regulatory network highlighting the interactions between miRNAs such as miR-132, miR-130, miR-203, and miR-221 and host molecules involved in inflammation, cell survival, proliferation, and immune responses such as NF-κB, VEGF, AKT, PI3K, TP53, and ERK.

At day 3 post-infection, network 2 centered on the PAX3-FOXO1 gene (Figure 3c). PAX3-FOXO1 has key targets such as IGF1R, TCF7L2, and ANXA1, suggesting its role in coordinating transcriptional and post-transcriptional regulatory processes. The analysis showed that PAX3-FOXO1 is modulated by multiple miRNAs, including miR-196b, miR-500, and miR-362-5p family members, which can target either PAX3-FOXO1 directly or its associated signaling nodes. Additionally, miR-1983 was identified as a regulator of PAX3-FOXO1. Similarly, at day 6 post-infection, network analysis highlighted the interactions among miRNAs, the PAX3-FOXO1 gene, and associated signaling molecules. In this network, PAX3-FOXO1 was shown to be modulated by several miRNAs, including miR-23a-3p, miR-221-3p, and miR-130a-3p. At day 9 post-infection, network 2 illustrated interactions between several miRNAs and genes such as CTNNB1, SMARCA4, and ESR1. Notably, ESR showed multiple regulatory interactions, including several miRNAs, including miR-664 and miR-199a-5p.

### 3.3. Pathway Analysis of POWV-Modulated miRNAs

**Figure 4:**
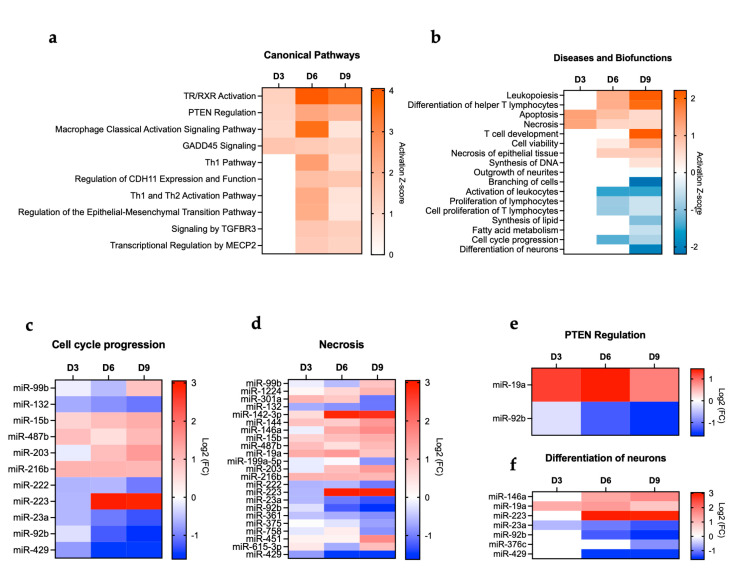
IPA. (**a**) Canonical pathways. (**b**) Diseases and biofunctions. miRNAs associated with (**c**) cell cycle progression, (**d**) necrosis, (**e**) PTEN regulation, and (**f**) differentiation of neurons.

IPA was also used to determine the top canonical pathways altered in the brain following POWV infection. Pathways such as thyroid hormone receptor and retinoid X receptor (TR/RXR) activation, phosphatase and tensin homolog (PTEN) regulation, macrophage classical activation, and growth arrest and DNA damage-inducible 45 (GADD45) signaling were activated as early as day 3 post-infection and persisted until day 9 post-infection. Notably, the highest activation of TR/RXR signaling and macrophage classical activation was observed at day 6 post-infection. Other pathways associated with Th1 activation and TGFBR signaling were among the activated canonical pathways at day 6 and 9 post-infection (Figure 4a). We also examined the enriched diseases and biofunctions. We observed the activation of biofunctions associated with an acutely activated immune system marked by processes such as leukopoiesis, differentiation of T lymphocytes, and T cell development. Notably, the heightened activation of immune-related pathways aligns with the increased viral load observed in the brain at days 6 and 9 post-infection. We also detected activation of cell death pathways such as apoptosis and necrosis. On the other hand, we observed the inhibition of other cellular processes such as synthesis of lipids, fatty acid metabolism, cell cycle progression, and differentiation of neurons (Figure 4b). We next delineated differentially expressed miRNAs associated with key pathways such as cell cycle progression, necrosis, PTEN regulation, and differentiation of neurons. We detected the decreased expression of several miRNAs that are related to cell cycle progression (miR-132, miR-222, miR-23a, miR-92b, and miR-429). In contrast, miR-15b, miR-487b, and miR-216b had increased expression (Figure 4c). Several miRNAs associated with necrosis such as miR-429, miR-92b, and miR-142-3p were also modulated (Figure 4d). Further, miRNAs associated with the PTEN signaling pathway (miR-19a and miR-92b) were differentially expressed in the brain following POWV infection (Figure 4e). Interestingly, we identified miR-223 overexpression in the brain, especially at day 6 and 9 post-infection (Figure 4f). miR-223 can modulate neuronal differentiation, neuroinflammation, and brain development, and its overexpression can delay neuronal maturation and impact neuronal differentiation [27,28].

**Figure 5:**
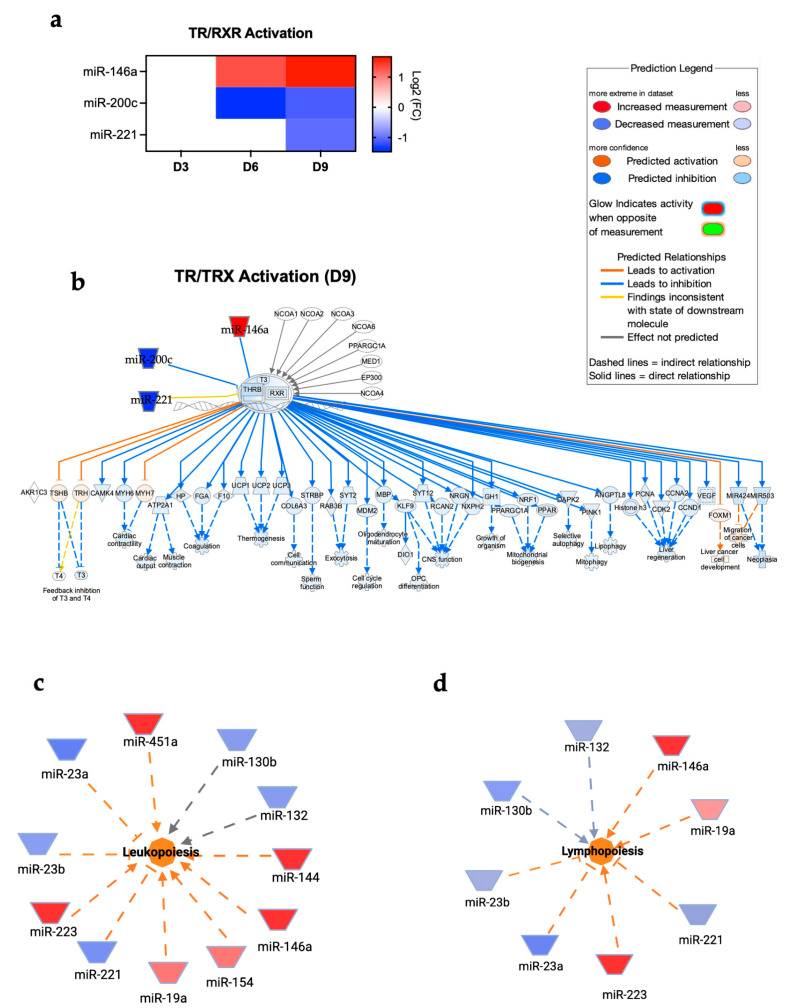
(**a**) Expression of miRNAs associated with TR/RXR pathways at day 3, 6, and 9 post-infection. (**b**) TR/RXR pathway at day 9 post-infection. Network diagrams of miRNAs associated with (**c**) leukopoiesis and (**d**) lymphopoiesis at day 9 post-infection. Red represents significantly upregulated; blue represents significantly downregulated. The color-coded arrows represent activation (orange), inhibition (blue), and unknown or indirect interactions (gray), with solid lines indicating confirmed interactions and dashed lines indicating predicted or inferred associations.

The TR/RXR signaling pathway plays a critical role in development, metabolism, and neural function in the central nervous system [29,30,31]. Our analysis showed the activation of TR/RXR in the brain following POWV infection. Dysregulated miRNAs involved in the TR/RXR signaling pathway are shown in Figure 5a. The miRNAs and targeted genes involved in the activation of the TR/RXR pathway are shown in Figure 5b. Our analysis also revealed an increase in leukopoiesis and lymphopoiesis with the highest activation observed at day 9 post-infection. Figure 5c shows the miRNAs involved in leukopoiesis. Notably, we detected the significant upregulation of miR-223, which is a master regulator of myeloid lineage differentiation, particularly granulopoiesis and monocyte/macrophage differentiation [32,33,34]. In the context of lymphopoiesis, miR-146a and miR-19a are known to play a role in regulating lineage commitment and lymphoid differentiation [35,36,37]. Both miR-146a and miR-19a were upregulated in POWV-infected brains (Figure 5d).

**Figure 6:**
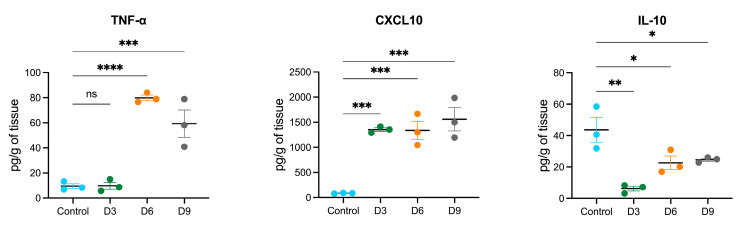
Cytokine and chemokine protein levels in the brain following POWV infection. Mice were inoculated with PBS (control) or POWV. Brain samples were collected at 3, 6, and 9 dpi. Cytokine and chemokine protein levels were measured by a multiplex immunoassay. Each data point represents an individual mouse. The middle horizontal bar indicates the mean, and error bars are SEM. Each analyte is plotted on an independent scale. *p*-values were calculated by one-way ANOVA followed by Dunnett’s multiple comparisons test (* *p* < 0.05; ** *p* < 0.01; *** *p* < 0.001; **** *p* < 0.0001; ns, non-significant). *n* = 3 for mock-infected; *n* = 3 for POWV-infected per time point).

We detected early upregulation of miR-19a that persisted until 9 dpi in POWV-infected brains. It has been shown that treatment of primary microglia cells with miR-19a results in the upregulation of cytokines such as tumor necrosis factor-α (TNF-α) and chemokines such as chemokine C-C motif ligand 2 (CCL2) and C-X-C motif chemokine ligand 10 (CXCL10) [38]. Another study showed that miR-19a plays a role in the suppression of interleukin-10 (IL-10) in peripheral dendritic cells [39]. Using a multiplex immunoassay, we measured the protein levels of TNF-α, CXCL10, and IL-10 in POWV-infected brains at 3, 6, and 9 dpi (Figure 6). We detected increased levels of TNF-α and CXCL10 starting at 3 dpi that became prominent by 9 dpi. On the other hand, we detected decreased levels of IL-10 in POWV-infected brains.

## 4. Discussion

Neurotropic viruses are capable of modulating miRNA expression to evade host immunity as well as to replicate efficiently in the host. This study is pioneering in investigating the miRNA signature in the brain following POWV infection. POWV infection in mice mimics human disease, thus making it an excellent model to understand the mechanisms that cause POWV encephalitis [40,41,42,43,44]. Using C57BL/6 mice, we demonstrated that POWV disseminates into the brain as early as 3 days post-infection with a progressive increase in viral RNA levels at days 6 and 9 post-infection. Using nCounter technology, we revealed the dysregulation of host miRNA in the brain during POWV infection. Notably, the viral replication trend was accompanied by the expanding miRNA response involved in immune activation, cell cycle control, and neurodegeneration.

We identified a progressive increase in both upregulated and downregulated miRNAs over the course of infection, suggesting an escalating host response that coincides with increased viral burden. Four miRNAs—miR-1983, miR-19a, miR-216b, and miR-500—were consistently dysregulated across all time points. Notably, miR-1983, miR-19a, and miR-216b were upregulated, while miR-500 was persistently downregulated. These miRNAs have been implicated in modulating inflammatory signaling and cellular homeostasis [45,46,47,48]. Consistent with our observation, the downregulation of miRNA-500 was observed following infection with MERS-CoV, SARS-CoV, and SARS-CoV-2 [49,50]. Additionally, miR-19a/b was shown to inhibit influenza A virus replication by targeting SOCS1 [51]. Studies have also demonstrated the abundant expression of miR-19a in exosomes derived from Hepatitis C virus-infected hepatocytes [46]. High levels of circulating miR-19a were detected in individuals with spinal cord injury with neuropathic pain [52]. Similarly, we showed increased expression levels of miR-19a at all time points. It was previously demonstrated that miR-19a promotes inflammation via toll-like receptor signaling, TNF signaling, and cytokine–cytokine receptor interactions [38]. Consistent with these results, the proteins levels of TNF-α and CXCL10 were significantly increased in POWV-infected brains.

Hierarchical clustering revealed two distinct clusters, with Cluster 1 comprising downregulated miRNAs such as miR-500 and miR-669m and Cluster 2 comprising upregulated miRNAs such as miR-15b and miR-1902. Interestingly, upregulation of miR-1902 was also reported in ZIKV-infected neurons [53]. Using network analysis, we identified key miRNAs such as miR-132 that functioned as a pivotal regulatory node. These findings are consistent with previous reports on the role of miR-132 during viral infections. For example, during influenza A virus infection, miR-132 accumulation in lung cells was observed [53]. It was also reported that miR-132 can enhance HIV replication in Jurkat cells [54] and is involved in ocular infection by herpes simplex virus [55].

We showed that TR/RXR signaling, PTEN regulation, macrophage classical activation, and GADD45 signaling were among the activated pathways in the brain following POWV infection. Notably, the activation of PTEN signaling aligns with the observed dysregulation of miRNAs such as miR-19a and miR-92b. We detected the upregulation of miR-19a in POWV-infected brains across all examined time points. Similarly, during viral myocarditis caused by Coxsackievirus B3, miR-19a was upregulated in the heart tissues of infected mice, facilitating biosynthesis and viral replication [45].

We uncovered the modulation of necrosis-associated miRNAs such as 142-3p especially at day 6 and 9 post-infection. Consistently, it was previously shown that miR-142-3p expression is dysregulated in human and mouse macrophages following infection by alphaviruses such as the Eastern equine encephalitis virus [56]. Our findings also underscored the role of miR-223 in immune trafficking and inflammatory balance during POWV infection [57]. Abnormal miR-223 expression is associated with inflammatory diseases such as rheumatoid arthritis and infections including HIV-1 and tuberculosis [32,33,34,58]. In the current study, we observed that miR-223 exhibited a biphasic expression pattern with decreased expression at 3 dpi followed by a significant upregulation at 6 and 9 dpi. These results suggest that the early suppression of miR-223 may allow for a robust initial inflammatory response, while its later induction likely acts to regulate excessive inflammation.

During DENV infection, miR-146a was upregulated with high expression in monocytes, the primary DENV target cells [12]. Studies have shown that miR-146a promotes DENV-2 viral replication by suppressing host IFN-β production [11]. Consistent with these results, we identified the increased expression of miR-146a in the brain, suggesting a crucial role of miR-146a during POWV infection. Additionally, during JEV infection, miR-15b was upregulated in glial cells and mouse brains, and in vitro overexpression of miR-15b enhanced JEV-induced inflammatory response. These studies have shown that JEV can directly target ring finger protein 125, resulting in increased RIG-I levels and higher production of pro-inflammatory cytokines [9,10,59]. Similarly, POWV infection increased the expression of miR-15b in the brain, particularly at day 6 and 9 post-infection.

Taken together, our study provides the first analysis of miRNA expression in the brain following POWV infection. The progressive modulation of miRNAs and associated pathways underscored the complexity of host–virus interactions in the CNS.

## Figures and Tables

**Table 1:** Primer sequences used for RT-qPCR.

	Forward Primer Sequence (5′->3′)	Reverse Primer Sequence (5′->3′)
POWV Probe	56-FAM/TGGCATCCG/Zen/AGAAAGTGATCCTGC/3IABkFQ	
POWV	GGCTGCAAATGAGACCAATTC	CAGCGACACATCTCCATAGTC

**Table 2:** Top up- and down-regulated DE miRNAs at 3, 6, and 9 dpi.

	D3	Log2FC	*p*-Value	D6	Log2FC	*p*-Value	D9	Log2FC	*p*-Value
Up-regulated	miR-1983	1.37	0.00010398	miR-223	3.05	1.43 × 10^−17^	miR-2135	2.92	1.12 × 10^−12^
	miR-19a	1.22	0.00210552	miR-142-3p	2.85	2.23 × 10^−15^	miR-2132	2.90	8.49 × 10^−13^
	miR-216b	1.14	0.00432204	miR-21	1.94	1.48 × 10^−9^	miR-223	2.88	9.2 × 10^−16^
	miR-496	1.14	0.00045631	miR-2135	1.82	0.0000118	miR-142-3p	2.71	5.13 × 10^−14^
	miR-1897-3p	1.12	0.00411598	miR-2132	1.66	0.000058	miR-21	2.44	2.76 × 10^−14^
Down-regulated	miR-706	−0.80	0.04311407	miR-500	−1.30	0.00039231	miR-23a	−1.29	0.0000176
	miR-196b	−0.93	0.00504502	miR-196b	−1.32	0.0000574	miR-182	−1.40	0.00022748
	miR-669m	−0.98	0.0471283	miR-182	−1.45	0.00011352	miR-145	−1.40	0.0000404
	miR-500	−1.06	0.00390904	miR-200c	−1.47	0.00185367	miR-141	−1.46	0.04859388
				miR-155	−3.28	0.00843883	miR-92b	−1.62	0.000000118

## Data Availability

The original contributions presented in the study are included in the article/Supplementary Materials; further inquiries can be directed to the corresponding author.

## Supplementary Materials

The following supporting information can be downloaded at https://www.mdpi.com/article/10.3390/v17101288/s1, Table S1: DE miRNAs with *p*-value < 0.05 and an absolute Log2 fold change greater than 0.75; Table S2: Time-specific DE miRNAs; Table S3: Network analysis by IPA.
